# Acquisition through Horizontal Gene Transfer of Plasmid pSMA198 by *Streptococcus macedonicus* ACA-DC 198 Points towards the Dairy Origin of the Species

**DOI:** 10.1371/journal.pone.0116337

**Published:** 2015-01-13

**Authors:** Konstantinos Papadimitriou, Rania Anastasiou, Eleni Maistrou, Thomas Plakas, Nikos C. Papandreou, Stavros J. Hamodrakas, Stéphanie Ferreira, Philip Supply, Pierre Renault, Bruno Pot, Effie Tsakalidou

**Affiliations:** 1 Laboratory of Dairy Research, Department of Food Science and Human Nutrition, Agricultural University of Athens, Iera Odos 75, 118 55, Athens, Greece; 2 Department of Cell Biology and Biophysics, Faculty of Biology, University of Athens, Panepistimiopolis, 157 01, Athens, Greece; 3 Genoscreen, Service of Research, Development and Innovation in Health and Environment, Campus de l’Institut Pasteur, 1 rue du Professeur Calmette, 59000, Lille, France; 4 Institut Pasteur de Lille, Center for Infection and Immunity of Lille (CIIL), F-59019, Lille, France; 5 Inserm U1019, F-59019, Lille, France; 6 CNRS UMR8204, F-59019, Lille, France; 7 Univ Lille de Nord France, F-59019, Lille, France; 8 INRA, UMR1319 Micalis, Jouy-en-Josas, F-78352, France; 9 AgroParisTech, UMR Micalis, Jouy-en-Josas, F-78352, France; Rockefeller University, UNITED STATES

## Abstract

**Background:**

*Streptococcus macedonicus* is an intriguing streptococcal species whose most frequent source of isolation is fermented foods similarly to *Streptococcus thermophilus*. However, *S. macedonicus* is closely related to commensal opportunistic pathogens of the *Streptococcus bovis*/*Streptococcus equinus* complex.

**Methodology/Principal Findings:**

We analyzed the pSMA198 plasmid isolated from the dairy strain *Streptococcus macedonicus* ACA-DC 198 in order to provide novel clues about the main ecological niche of this bacterium. pSMA198 belongs to the narrow host range pCI305/pWV02 family found primarily in lactococci and to the best of our knowledge it is the first such plasmid to be reported in streptococci. Comparative analysis of the pSMA198 sequence revealed a high degree of similarity with plasmids isolated from *Lactococcus lactis* strains deriving from milk or its products. Phylogenetic analysis of the pSMA198 Rep showed that the vast majority of closely related proteins derive from lactococcal dairy isolates. Additionally, cloning of the pSMA198 ori in *L. lactis* revealed a 100% stability of replication over 100 generations. Both pSMA198 and the chromosome of *S. macedonicus* exhibit a high percentage of potential pseudogenes, indicating that they have co-evolved under the same gene decay processes. We identified chromosomal regions in *S. macedonicus* that may have originated from pSMA198, also supporting a long co-existence of the two replicons. pSMA198 was also found in divergent biotypes of *S. macedonicus* and in strains isolated from dispersed geographic locations (e.g. Greece and Switzerland) showing that pSMA198’s acquisition is not a recent event.

**Conclusions/Significance:**

Here we propose that *S. macedonicus* acquired plasmid pSMA198 from *L. lactis* via an ancestral genetic exchange event that took place most probably in milk or dairy products. We provide important evidence that point towards the dairy origin of this species.

## Introduction

Lactic acid bacteria (LAB) form the most important group of microorganisms used in food fermentations. Several LAB species have a long history of unproblematic use as starters and they are thus considered as safe including a number of probiotic strains that may provide the consumers with health benefits [1]. However, food-related LAB are often phylogenetically related to commensal LAB that may be opportunistic or even invasive pathogens. The most striking example of this situation is probably the genus *Streptococcus*, in which only one species, *Streptococcus thermophilus* is considered safe. Since centuries, *S. thermophilus* is a dairy starter frequently consumed by humans world-wide. We now know that this species is phylogenetically related to notorious streptococcal pathogens such as Group A and Group B streptococci, as well as to *Streptococcus pneumoniae*. Studies of the *S. thermophilus* genome, however, have revealed extensive gene decay, leading to the loss of streptococcal pathogenic determinants during its adaptation to milk [2,3]. In addition, the species was shown to have acquired genes from species like *Lactobacillus delbrueckii* subsp. *bulgaricus* with whom it often shares a symbiotic relationship in the dairy environment [4]. These attributes along with the long empiric experience of its safe use support the benign nature of *S. thermophilus* [5].

Apart from *S. thermophilus*, certain members of the *Streptococcus bovis*/*Streptococcus equinus* complex (SBSEC), i.e. *Streptococcus macedonicus* and *Streptococcus infantarius*, have also been associated with fermented foods [6,7]. After its original description *S. macedonicus* has been identified in a wide range of dairies around the world and current studies indicate that milk and its products represent its primary ecological niche [6]. Similarly to *S. macedonicus*, strains of *S. infantarius* have been isolated from the food environment [8,9]. Both species are clearly related to the other members of the SBSEC, such as the commensals *Streptococcus gallolyticus* and *Streptococcus pasteurianus* that are associated with endocarditis, bacteremia and colon cancer [10,11]. *Streptococcus infantarius* has also been implicated with human disease [12], while there are only few studies suggesting the presence of *S. macedonicus* in cases of endocarditis [13,14]. Analysis of the recently sequenced genomes of *S. macedonicus* ACA-DC 198 (isolated from traditional Greek Kasseri cheese) and of *S. infantarius* CJ18 (isolated from suusac, a type of traditionally Kenyan fermented camel milk) has facilitated the assessment of the adaptation of these strains to milk [12,15]. Both strains present traits of adaptation to the dairy environment. For example, *S. macedonicus* appears to have evolved under selective pressures that favored genome decay and gene loss processes, resembling the evolution pattern of *S. thermophilus* [16]. The strain has lost important genes present in the rumen bacterium *S. gallolyticus* that are involved in complex plant carbohydrate catabolism and in the detoxification of toxic substances met in the gastrointestinal tract of herbivores (e.g. tannins and gallic acid), thus indicating deviation from this environment. Furthermore, the strain owns a second gene cluster for lactose catabolism unique within the SBSEC, a proteolytic system capable of degrading milk proteins and enhanced resistance to phages like that of known dairy starters. Analogous dairy adaptations were described for *S. infantarius* CJ18 [17]. Perhaps the most convincing evidence suggesting milk as the ecological niche of the two strains is the detection of interactions through horizontal gene transfer (HGT) with the dairy *Lactococcus lactis* and *S. thermophilus*. Such HGT events seem to be specific for *S. macedonicus* and *S. infantarius* and to be absent from *S. gallolyticus* and *S. pasteurianus.* Events like these in the evolutionary history of the two species suggest a longstanding relationship with milk and fermented dairy products, even though their actual presence in these niches was only recently confirmed [6,9]. Among the most common HGT mechanisms is conjugation which involves the transfer of DNA by plasmids through plasmid encoded cell-to-cell junctions [18]. Conjugation may also result in the transfer of plasmids that are not conjugative but they carry the necessary mobilization genes.

In this study we attempted to clarify the route of the original acquisition of pSMA198, the first plasmid isolated from *S. macedonicus* ACA-DC 198. Acquisition of pSMA198 by *S. macedonicus* may represent an apparent HGT event in the evolutionary history of the strain. We assessed the hypothesis that the plasmid was originally acquired by *S. macedonicus* from *L. lactis* and that this acquisition took place in the dairy environment. We also tested the replication efficiency of pSMA198 in *L. lactis*. Finally, we investigated whether lactococcal sequences found in the chromosome of *S. macedonicus* ACA-DC 198 could have originated from plasmid pSMA198. Our findings point towards the dairy origin of *S. macedonicus* ACA-DC 198.

## Materials and Methods

### Bacterial strains and culture conditions

*Streptococcus macedonicus* ACA-DC 198 was originally isolated from traditional Greek Kasseri cheese [19]. The strain was routinely cultured in M17 (Oxoid Ltd., Basingstoke, Hampshire, United Kingdom) supplemented with either 1% (w/v) lactose (LM17) or 0.5% (w/v) glucose (GM17). *Lactococcus lactis* subsp. *cremoris* MG1363 was grown in GM17. Stocks of these bacteria were stored at −80°C in cryovials filled with 1 ml of GM17 supplemented with 20% (v/v) glycerol. *Escherichia coli* HST08 strain (Stellar Competent Cells, Clontech Inc., Mountain View, CA) was grown in Luria Bertani (LB) medium under aerobic conditions. Antibiotics and other supplements were added in the media at appropriate concentrations as indicated below.

### Sequencing and annotation of pSMA198

The sequence of pSMA198 was identified during the genome sequencing of *S. macedonicus* ACA-DC 198 as described previously [15]. In brief, total DNA of *S. macedonicus* was subjected to pyrosequencing according to the manufacturer’s instructions on a 454 GS-FLX Titanium sequencer (Roche Diagnostics, Basel, Switzerland) (Genoscreen, Lille, France). A round of shotgun pyrosequencing was followed by a 3-kb paired-end pyrosequencing. The acquired sequences were assembled using Newbler into 7 scaffolds, 5 of which could be subsequently aligned against a *Nhe*I genomic optical map of *S. macedonicus* (OpGen Technologies, Inc., Madison, WI). Further analysis supported that one of the two unaligned scaffolds was a plasmid. The complete sequence of pSMA198 was finally acquired at a >100X coverage through hybrid assembly of the pyrosequencing reads with reads obtained after an additional round of Hiseq2000 (Illumina, San Diego, CA) sequencing (Fidelity Systems, Inc., Gaithersburg, MD) (accession number: HE613570).

Gene prediction and annotation of the plasmid was performed using two *ab initio* predictor pipelines, RAST [20] and BaSys [21]. We also employed manual WU-Blast similarity searches for the detailed functional annotation of deduced proteins [22]. Pseudogenes were considered on the basis of presence or absence of ribosome-binding sites (RBS), of the coding sequence length and of frame shifts resulting in split genes. The origin of replication (*ori*) and the origin of mobilization (*oriT*) were identified based on previously determined sequences in plasmids related to pSMA198.

### Nucleotide and protein analysis and phylogenetic analysis

In addition to WU-BlastN and WU-BlastP searches, nucleotide and protein sequences were aligned using ClustalW [23]. Miscellaneous features within the alignments (i.e. direct repeats, inverted repeats and other important sequences) were determined manually based on the available literature. We used InterProScan [24] to detect motifs within protein sequences. For phylogenetic analysis multiple sequence alignments were curated with Gblocks [25] and maximum likelihood trees were generated with the PhyML algorithm [26] as implemented in the Phylogeny.fr pipeline [27]. Branch support values were calculated with the χ^2^ parametric approximate likelihood-ratio test (aLRT) [28]. Graphical representation of the phylogenetic tree was carried out with TreeDyn [29]. Comparative analysis of pSMA198 to other plasmids was performed using Circoletto [30] and Kodon software (Applied Maths N.V., Sint-Martens-Latem, Belgium).

### Construction of an *E. coli*—*S. macedonicus* shuttle vector based on the pSMA198 replicon

Primers used for the construction of the *E. coli*—*S. macedonicus* shuttle vector are presented in Table 1. Total DNA was extracted using the GenElute Bacterial Genomic DNA Kit (Sigma-Aldrich, St Louis, MO) and plasmids were isolated with the NucleoSpin Plasmid kit (Macherey-Nagel GmbH and Co. KG, Düren, Germany). PCR products were purified from agarose gels using the NucleoSpin Gel and PCR Clean-up kit (Macherey-Nagel GmbH). The entire pSMA198 replication backbone (*ori*—*rep*—*orfX*) was cloned into the pre-linearized pUC19 plasmid using the ligation-free In-Fusion HD Cloning Kit (Clontech Inc.) according to the manufacturer’s instructions. In brief, the replication backbone of pSMA198 was amplified by PCR using primers pr2Fn/pr2R designed to hybridize at the ends of both the replication backbone of pSMA198 and the linearized pUC19 vector. The PCR product was gel purified and then incubated along with pUC19 in the In-Fusion cloning reaction for 15 min at 50°C. Five microliters of the reaction was used to transform *E.coli* Stellar chemically competent cells. The cells were plated on LB agar plates containing 100 μg/ml ampicillin. Forty microliters of a 20 mg/ml X-gal solution was spread on the surface of the plates for blue—white selection. Colony PCR was performed on white colonies to verify cloning of the right insert. Two colonies were selected for plasmid isolation. The erythromycin resistance gene was amplified by PCR from plasmid pGh9:IS*S1* [31] using primers ermF1/ermR1. The plasmids containing the pSMA198 replicon were digested with the *Pst*I enzyme (New England Biolabs Inc., Beverly, MA) and the erythromycin resistance gene was cloned in this position. The cloning reactions were used to transform *E. coli* Stellar cells. Transformed cells were plated on LB agar containing 200 μg/ml erythromycin. Colony PCR was employed to screen for clones containing both inserts. Two plasmids were sequenced in the entire region spanning the hybridization positions of M13F and M13R primers to verify that no mutations were introduced in the two inserts during the cloning procedures. The sequence of the plasmid assigned as pORI198 was deposited in EMBL database under accession number HG974440.

**Table 1:** List of primers used in this study.

**Primer**	**Sequence** **^a^**
pr2Fn	GAGCTCGGTACCCGGGGATCATGGACGAGCCACTCGTATC
pr2R	CAGGTCGACTCTAGAGGATCCATTCATCTATTTCTCCCTCTCTTC
ermF1	TGGATCCTCTAGAGTCGACCTGCAGACGTATATAGATTTCATAAAGTC
ermR1	TTACGCCAAGCTTGCATGCCTGCAGCATTCCCTTTAGTAACGTGTAAC
repF	GAAATCAACGCCCATACGTC
repR	TATCGTCTGCACACCGTTTC
tufAF	GGTAGTTGTCGAAGAATGGAGTGTGA
tufAR	TAAACCAGGTTCAATCACTCCACACA
mcdMF	CGGAATTCAGTTCTTTCTACGG
mcdMR	GCTTCACCAATAAGCGTTCC
reb1F	GGACGGAGCATTGACTCTATTG
reb1R	CTGTTCCTGCAAATTTTCCAAC
mobb1F	ATTGGGTTCTGATTTTGGAAGG
mobb1R	CACCTAATCCAAGAACGACTGC
M13F	GTAAAACGACGGCCAGT
M13R	CAGGAAACAGCTATGAC

^a^All primers were designed during this study except from primers tufAF/tufAR that were designed in [55] and primers M13F/M13R that hybridize with the pUC19 backbone.

### Cloning pORI198 in *L. lactics* subsp. *cremoris* MG1363 and assessing the stability of the plasmid in this host

Electrocompetent cells of *L. lactis* MG1363 were prepared as described previously [32]. One microgram of pORI198 was used during electroporation. *Lactococcus lactis* cells were plated on GM17 agar containing 1 μg/ml erythromycin at 37°C. Colonies were screened for the pORI198 plasmid using primers M13F/M13R. One positive colony was grown overnight in 5 ml GM17 broth containing 1 μg/ml erythromycin and cells were stored at −80°C as described above.

*Lactococcus lactis* containing the pORI198 plasmid was subcultured once in GM17 broth containing 1 μg/ml erythromycin. Cells were washed twice in Ringer solution to remove any traces of erythromycin and then inoculated in GM17 broth without erythromycin. The strain was subcultured every 12 h so as to multiply for approximately 10 generations within this timeframe. The strain was propagated for a total of 100 generations and every 20 generations samples were serially diluted and plated on GM17 agar containing or not 1 μg/ml erythromycin to assess the stability of the plasmid. Three independent experiments were performed. Differences between the numbers of cells growing in the presence or absence of erythromycin were assessed with Student’s t-test for p<0.05.

### Quantification of the pSMA198 or pORI198 plasmid copy number (PCN) in the relevant hosts and assessment of the presence of pSMA198 in strains of *S. macedonicus*

All primers used to quantify the PCN of the two plasmids are presented in Table 1. The PCN of pSMA198 in *S. macedonicus* and of pORI198 in *L. lactis* was determined based on the qPCR method described by Skulj et al. (2008) [33] and as performed previously [34]. In brief, total DNA was extracted as described by Leenhouts et al. [35]. Internal fragments of the single copy genes *mcdM* and *tufA* were used as chromosomal references for *S. macedonicus* and *L. lactis*, respectively. For PCN determination an internal segment of the single copy plasmid gene *rep* of both plasmids was amplified. qPCR was performed on a MX3005P (Stratagene, La Jolla, CA) using the KAPA SYBR FAST qPCR Kit (Kapa Biosystems, Inc. Woburn, MA) according to the manufacturer’s instructions. The PCN of pSMA198 was determined after three independent experiments.

For the assessment of the presence of pSMA198 in different strains of *S. macedonicus*, cultures were grown to stationary phase before total DNA isolation with the GenElute Bacterial Genomic DNA Kit. The presence/absence of pSMA198 was verified by PCR using primers reb1F/reb1R with the Taq PCR 2X ReadyMix kit (Kapa Biosystems, Inc.). To avoid any false positive results the presence of the plasmid was also verified with primers mobb1F/mobb1R.

## Results and Discussion

### pSMA198 belongs to the narrow host range pCI305/pWV02 family of lactococcal plasmids

**Figure 1:**
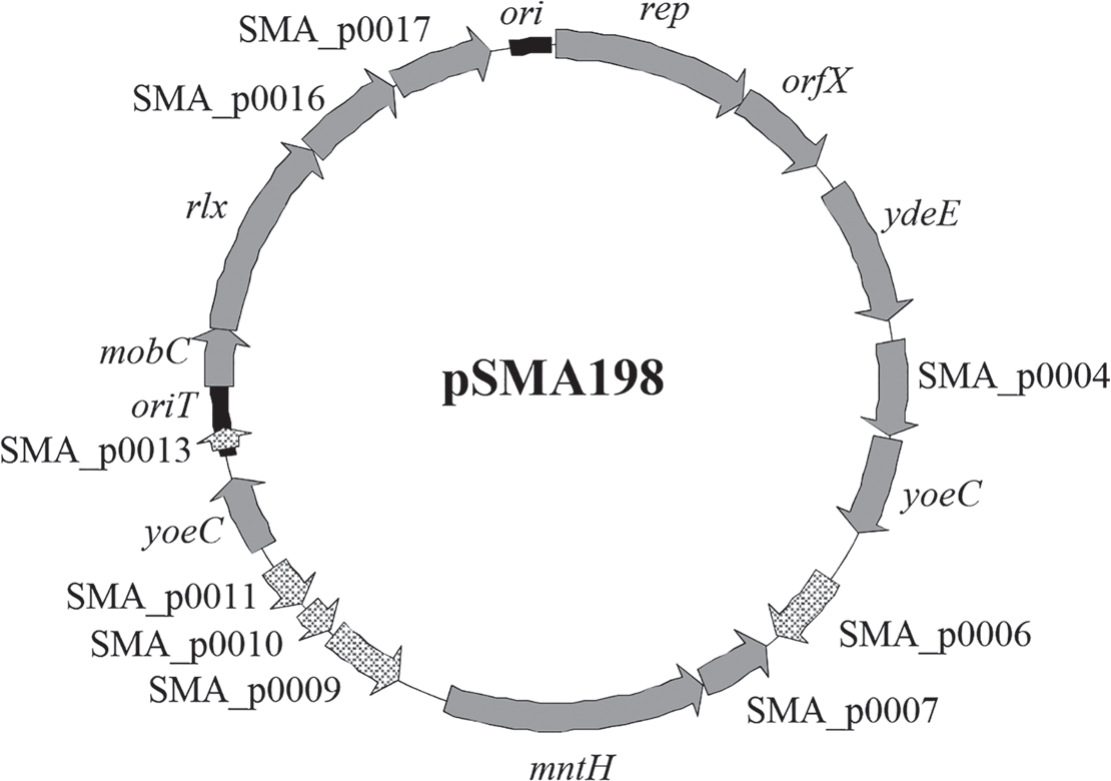
Annotated map of pSMA198. Black boxes indicate the position of *ori* and *oriT*. Solid grey arrows indicate positions of functional genes. Remaining light grey arrows indicate positions of predicted pseudogenes.

**Figure 2:**
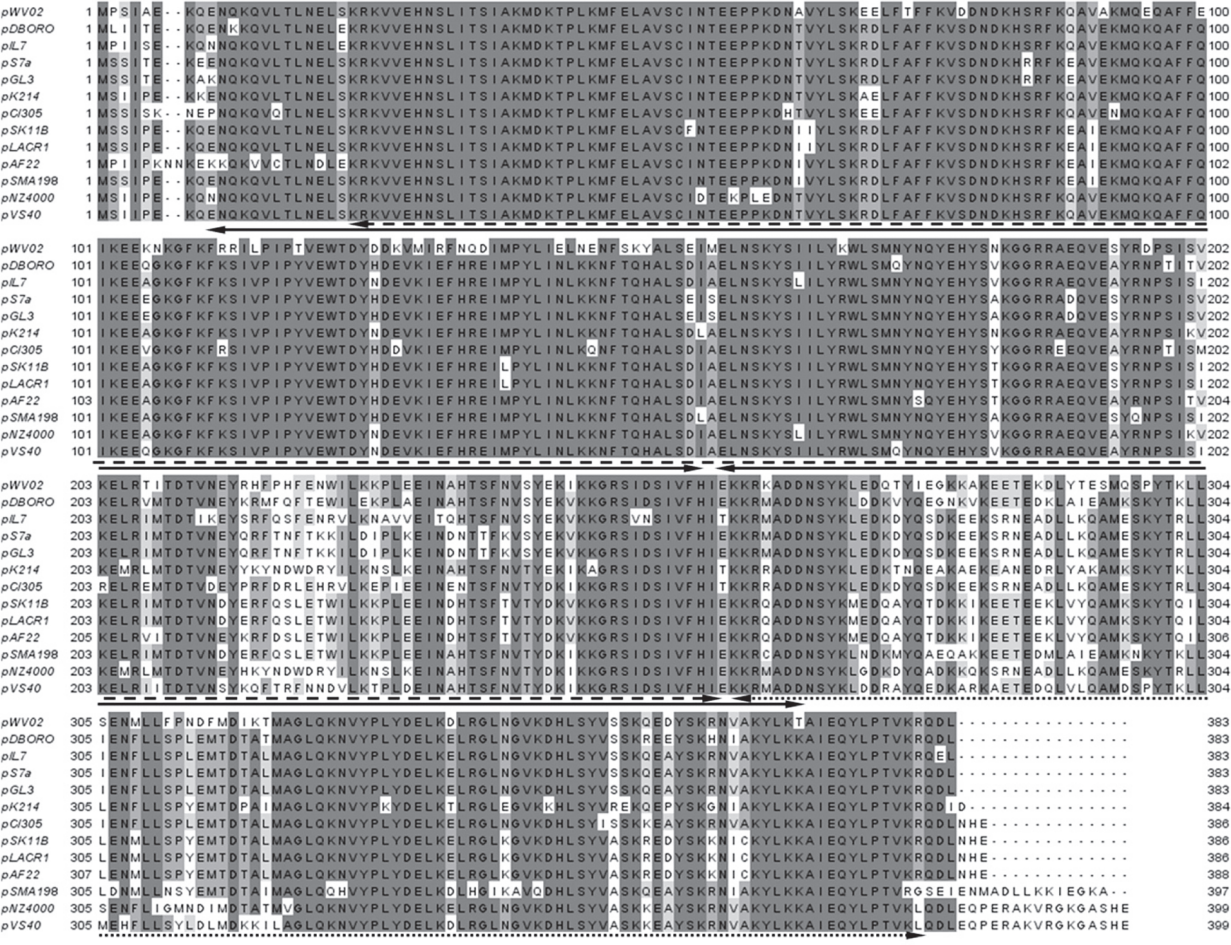
Multiple sequence alignment of RepB sequences relevant to the respective protein of pSMA198. The alignment was performed with ClustalW [23]. Double-headed arrows indicate positions of protein sequence signatures as determined by InterProScan [24], as follows: initiator Rep protein (dashed line), *L. lactis* RepB C-terminal (dotted line) and two consecutive winged helix-turn-helix transcription DNA-binding repressor (solid line).

*Streptococcus macedonicus* ACA-DC 198 carries a novel plasmid of 12,728 bp assigned as pSMA198 [15]. The plasmid has a 35.0% G+C content, which is lower than that of the *S. macedonicus* chromosome (37.6%), suggesting that it may have been acquired from another organism. Overall, 17 CDSs were identified on pSMA198 (Fig. 1 and Table 2). The first gene codes for a replication initiation protein (Rep). The *rep* gene showed 87% of DNA sequence identity (e-value 2.0e^−196^) with the respective gene found on plasmid 1 (pl1) of *L. lactis* subsp. *cremoris* SK11 [36]. Among the best WU-Blast hits of Rep we identified the RepB proteins of the pCI305 and the pWV02 plasmids (78% identity, e-value 1.7e^−161^ and 75% identity, e-value 5.9e^−152^, respectively) that are the prototypes of the pCI305/pWV02 family of the lactococcal theta-replicating plasmids [37–39]. Multiple sequence alignment of the top Rep WU-Blast hits, including the pCI305 and pWV02 RepB proteins, revealed a high degree of conservation among them (Fig. 2). Analysis of these proteins with InterProScan determined four Pfam sequence signatures corresponding to the initiator Rep protein (Rep_3, PF01051), the *L. lactis* RepB C-terminal protein (L_lactis_RepB_C, PF06430) and two consecutive winged helix-turn-helix transcription DNA-binding domains (Wing_hlx_DNA_bd, G3DSA:1.10.10.10). The first signature is typically shared by the theta replicon-type Rep proteins [40], while according to our analysis its combination with the second signature seems to be specific and characteristic of the RepB proteins of the pCI305/pWV02 family. The product of the gene *orfX*, found immediately downstream of *rep*, has been suggested to participate in the control of the PCN [39]. OrfX is significantly less conserved than Rep (data not shown). This is not completely unexpected, as in some plasmids *orfX* is absent, indicating that it may not be necessary for the replication of the plasmids carrying it [39].

**Table 2:** Annotated features of pSMA198.

**locus_tag**	**gene**	**size nt**	**Best WU-Blastn hit (locus or locus_tag/ organism/ identity/ e-value)**	**Protein function**
SMA_p0001	*rep*	1194	LACR_A06/ *Lactococcus lactis* subsp. *cremoris* SK11 plasmid 1/ 87%/ 2.0e^−196^	Initiator RepB protein
SMA_p0002	*orfX*	585	SPJ1_2277/ *Streptococcus parauberis*KRS-02083/ 91%/ 2.1e^−101^	Replication associated protein
SMA_p0003	*ydeE*	858	I571_01319/ *Enterococcus durans*ATCC 6056/ 99%/ 1.0e^−184^	AraC family transcriptional regulator
SMA_p0004	-	582	OGW_05102/ *Enterococcus faecium*EnGen0004/ 99%/ 8.2e^−122^	Integral membrane protein
SMA_p0005	*yoeC*	591	OIU_05588/ *Enterococcus faecium*EnGen0039/ 98%/ 5.3e^−121^	Integrase/recombinase plasmid associated
SMA_p0006	-	459	CAC42047/ *Listeria innocua* Clip11262 pLI100/ 99%/ 4.2e^−94^	Putative pseudo
SMA_p0007	-	438	LACR_D31/ *Lactococcus lactis* subsp. *cremoris* SK11 plasmid 4/ 99%/ 5.6e^−90^	Universal stress protein family
SMA_p0008	*mntH*	1578	SPJ2_0535 / *Streptococcus parauberis*KRS-02109/ 99%/ 0.0	Manganese transport protein MntH
SMA_p0009	-	480	llmg_pseudo_13/ *Lactococcus lactis* subsp. *cremoris* MG1363 pseudogene/ 97%/ 3.7e^−214^	Putative pseudo
SMA_p0010	-	195	llmg_pseudo_13/ *Lactococcus lactis* subsp. *cremoris* MG1363 pseudogene/ 97%/ 3.7e^−214^	Putative pseudo
SMA_p0011	-	276	llmg_pseudo_13/ *Lactococcus lactis* subsp. *cremoris* MG1363 pseudogene/ 97%/ 3.7e^−214^	Putative pseudo
SMA_p0012	*yoeC*	465	OGQ_02346 / *Enterococcus faecium*EnGen0017/ 98%/ 2.5e^−93^	Integrase/recombinase plasmid associated
SMA_p0013	-	132	pIL7_28/ *Lactococcus lactis* subsp. *lactis* IL594 plasmid pIL7/ 84%/ 1.4e^−13^	Putative pseudo
SMA_p0014	*mobC*	366	HMPREF9519_01999/ *Enterococcus faecalis* TX1346/ 89%/ 6.7e^−61^	Mobilization protein
SMA_p0015	*rlx*	1233	CI5MOBPRO/ *Lactococcus lactis* subsp. *cremoris* UC503 pCI528/ 99%/ 3.8e^−268^	Mobilization protein
SMA_p0016	-	627	ENT_30400 / *Enterococcus* sp. 7L76/ 96%/ 7.1e^−124^	Conserved hypothetical protein
SMA_p0017	-	603	BN193_11500/ *Lactococcus raffinolactis* 4877/ 99%/ 4.0e^−125^	Fic family protein

Other potentially interesting proteins encoded by pSMA198 are a transcriptional regulator of the AraC family (YdeE), a universal stress protein (SMA_p0007) and a manganese transport protein (MntH). The fourth gene codes for an integral membrane protein of unknown function, while the fifth and the twelfth genes are both coding for an integrase/recombinase plasmid-associated YoeC protein. Five ORFs are putative pseudogenes. Four (SMA_p0006, SMA_p0009, SMA_p0010 and SMA_p0011) consist of fragments of genes coding for transposase elements. The remaining pseudogene SMA_p0013 is devoid of an RBS sequence. It is also noteworthy that, according to current annotations, analogs of all these pseudogenes could be identified in a potentially functional form in different lactococcal plasmids related to pSMA198 (e.g. SMA_p0006 in pIL5, SMA_p0009, SMA_p0010 and SMA_p0011 in pGdh442 and SMA_p0013 in pCI605, data not shown). These findings indicate that pSMA198 has been subjected to gene decay processes. The last four genes of the plasmid are probably dedicated to the mobilization of the plasmid. Apart from *mobC* and *rlx* coding for mobilization proteins, two additional genes (SMA_p0016 and SMA_p0017) encode a conserved hypothetical protein and a Fic family protein, respectively. Genes like SMA_p0017 are often found in the same context with mobilization-associated genes and are probably implicated in cell cycle regulation [41].

**Figure 3:**
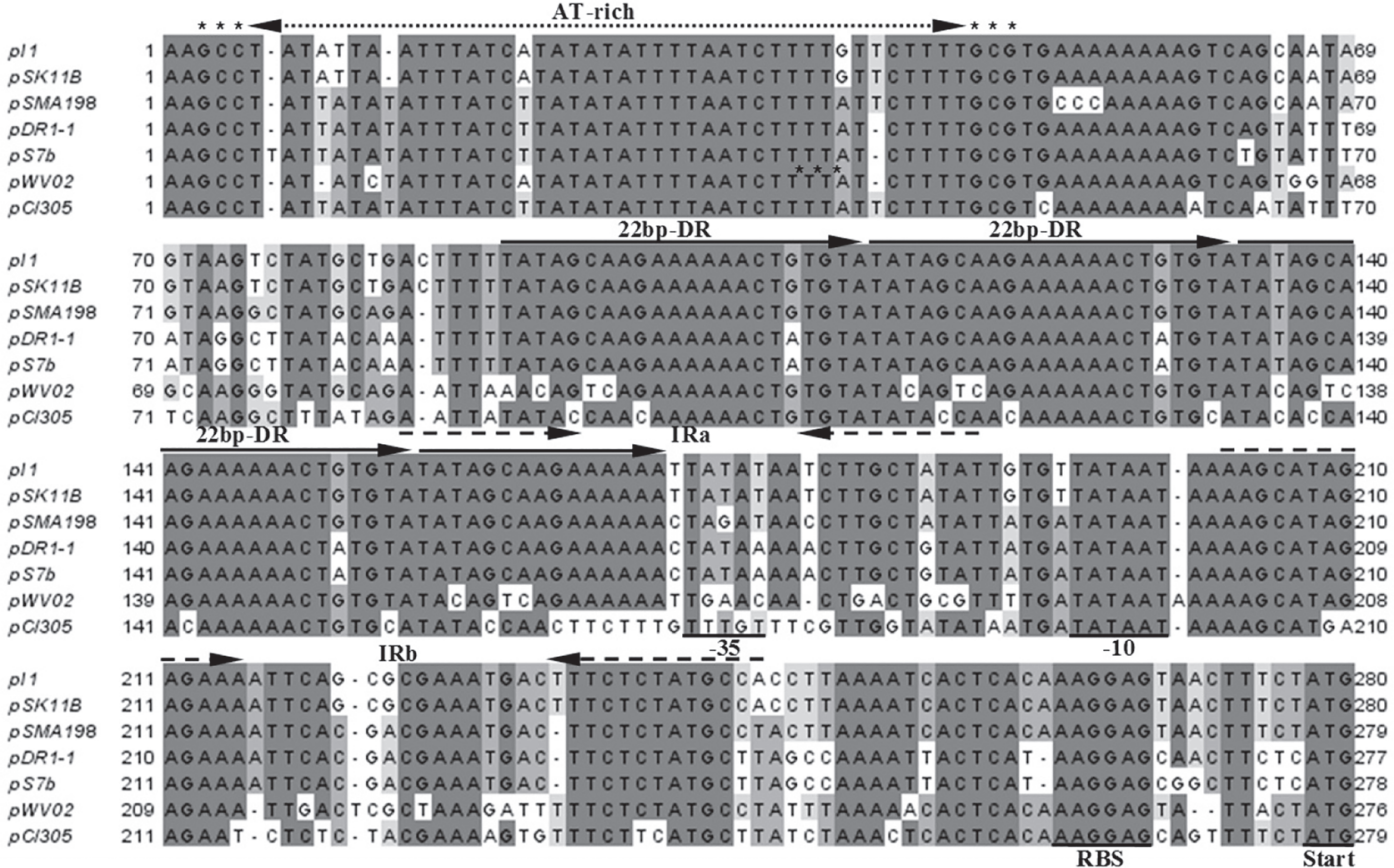
Multiple sequence alignment of *ori* of pSMA198 and its related plasmids. The alignment was performed using ClustalW [23]. Arrows indicate the position of the AT-rich region, the 22-bp direct repeat (DR) iterons and two inverted repeats (IR). The predicted leader region (−35, −10 and the RBS) and the start codon of the *rep* gene are underlined.

Subsequently, we looked upstream of the *rep* gene in an attempt to identify the origin of replication (*ori*) of pSMA198. WU-Blastn similarity searches and multiple sequence alignment directed us towards a pCI305/pWV02 type of *ori* (Fig. 3). Indeed, we determined a segment spanning 242 nucleotides that contains an AT-rich region, three and a half direct repeats (DRs) of 22-bp iterons and two inverted repeats (IRs). The pattern of the pSMA198 *ori* along with the similarity of its Rep with the lactococcal RepB shows that pSMA198 is certainly a member of the pCI305/pWV02 family of replicons, which are normally found in *Lactococcus* species [37–39].

**Figure 4:**
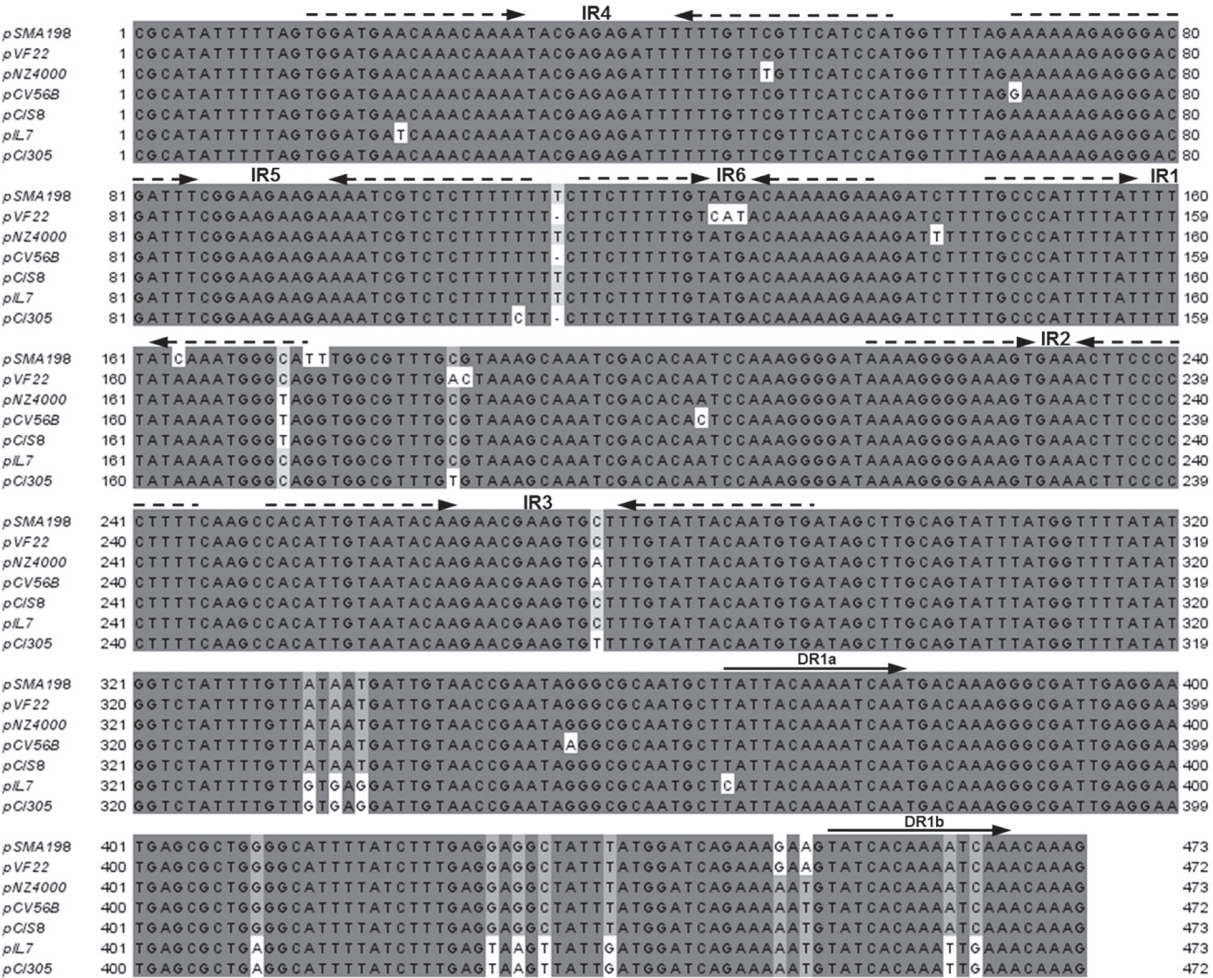
Multiple sequence alignment of *oriT* of pSMA198 and its related plasmids. The alignment was performed using ClustalW [23]. Arrows indicate positions of the six inverted repeats (IR) and the two directed repeats (DR).

In addition, even though pSMA198 is not a self-transmissible plasmid, a *cis*-acting origin for transfer (*oriT*) that would allow its mobilization in the presence of a true conjugative plasmid was also predicted upstream of *mobC* (Fig. 4). The *oriT* exhibited a region of six consecutive IRs and two DRs. Eight bases after the end of IR3 we determined an identical nick site to those previously proposed for plasmids pS7a and pS7b found in *L. lactis* subsp. *lactis* biovar. diacetylactis S50 isolated from butter [42]. Once more, these structures are highly conserved among several lactococcal plasmids including pCI305 [42,43].

### pSMA198 was acquired by *S. macedonicus* from *L. lactis* probably in the dairy environment

**Figure 5:**
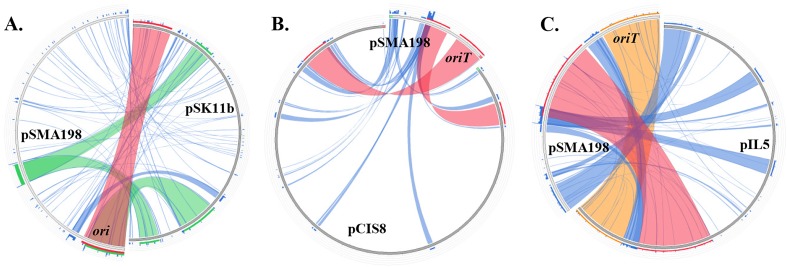
Sequence alignment of pSMA198 against pSK11b (A), pCIS8 (B) and pIL5 (C) represented in a circular fashion using Circoletto [30]. Local alignments produced by BLAST are presented using ribbons whose color corresponds to four quartiles of the alignment’s bitscore (red: top 25%, orange: second 25%, green: third 25% and blue: worst 25%). The positions of pSMA198 *ori* or *oriT* were added in order to aid orientation.

The relation of pSMA198 to other plasmids was further investigated (Fig. 5). WU-Blastn searches supported that the closest plasmids were of lactococcal origin. Top hits were aligned against pSMA198 in a circular manner using Circoletto. We initiated our search with the pSMA198 *ori* and *oriT* that showed highest identity to the corresponding features of plasmids pSK11b (92% identity, e-value 8.4e^−44^) and pCIS8 (98% identity, e-value 5.1e^−95^), respectively. Both pSK11b and pCIS8 have been isolated from *L. lactis* subsp. *cremoris* strains used as dairy starters [44,45]. We also looked for the plasmid that would have the highest identity with the complete sequence of pSMA198. The plasmid identified was pIL5 that has been isolated from the cheese starter *L. lactis* subsp. *lactis* IL594 [43]. pIL5 showed 97% identity (e-value 0.0) with a query coverage of approximately 30% of a central part between *ori* and *oriT* of plasmid pSMA198 (Fig. 5). It should be emphasized that apart from the close similarity hits mentioned above, the overriding majority of the top hits for the different features annotated on pSMA198 at the protein and nucleotide level originated from *L. lactis* dairy strains. For example, nine out of ten top hits for the replication backbone originated from strains isolated from milk or its products. Therefore, it is most likely that the original donor of pSMA198 was a dairy *L. lactis* strain.

To provide further evidence for this hypothesis we performed a phylogenetic analysis of the RepB protein (S1 Fig.). The pSMA198 Rep sequence was placed within a branch in which 28 proteins out of 32 originated from dairy lactococcal strains. Only four proteins within the branch could be traced back to non-dairy species. Furthermore, the fact that non-lactococcal proteins were limited to 47 out of the 200 RepB included in the phylogenetic tree is in line with a relatively restricted dispersion of pCI305/pWV02 plasmids to species other than lactococci. This is in accordance with the experimental data that suggested a narrow host range nature of these plasmids [37,46]. Another potential explanation of the high prevalence of lactococcal sequences in the phylogenetic tree could be that the sequence database is biased due to the high number of sequenced *L. lactis* strains especially of dairy origin. Even though we acknowledge this possibility, there is currently no direct way to validate or refute it. Nevertheless, the absence of pCI305/pWV02 plasmids from the high number of sequenced LAB strains other than *L. lactis* is also an indirect indication of the lactococcal origin of this family of replicons.

**Figure 6:**
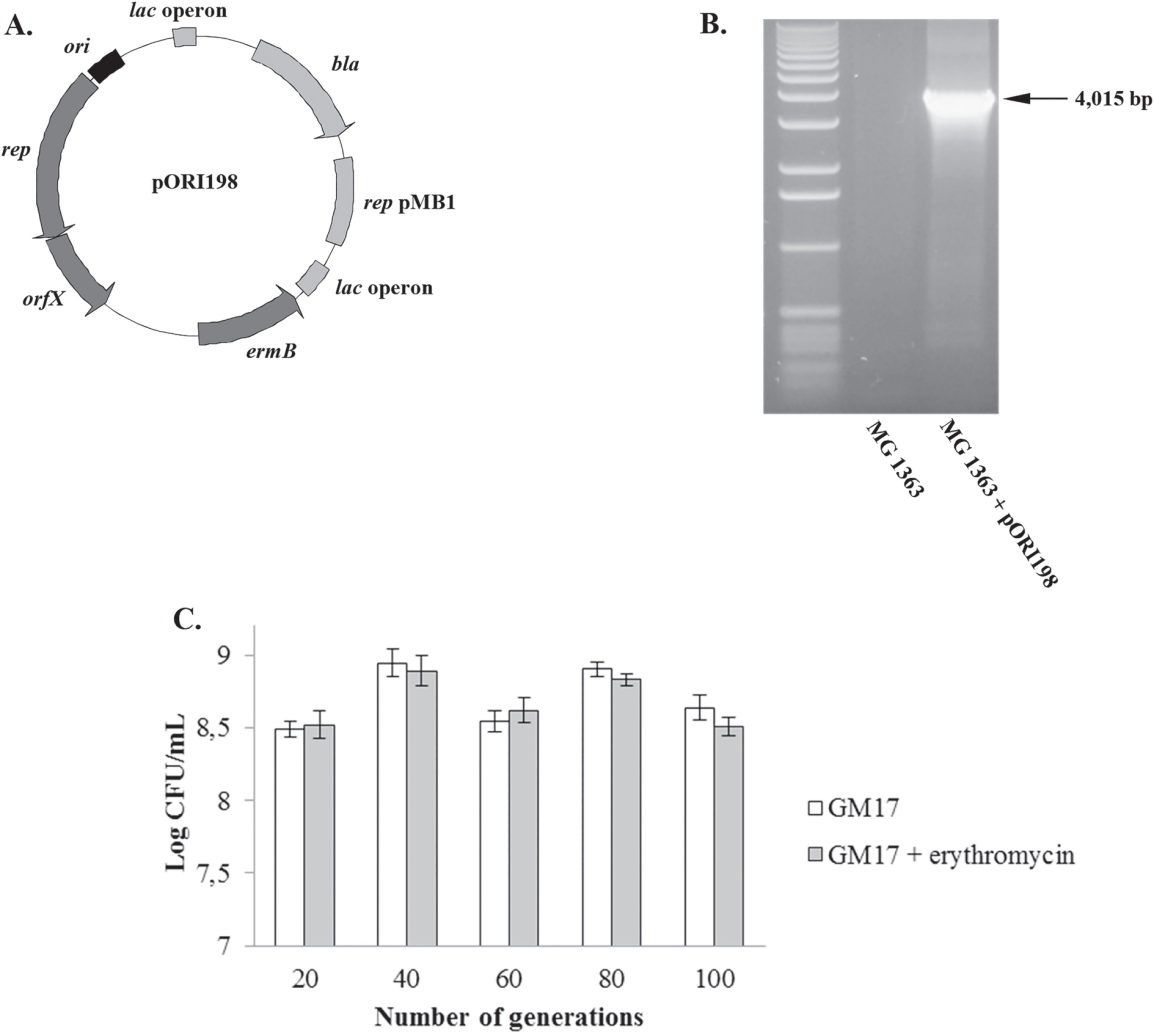
Cloning plasmid pORI198 in *L. lactis* MG1363 and assessing its stability in this host. Annotated map of pORI198 (A). The black box and the dark grey arrows indicate the position of the cloned pSMA198 replication backbone (*ori*—*rep*—*orfX*). The light grey box and arrows indicate the position of the pUC19 cloning vector. Cloning pORI198 in *L. lactis* (B). The presence of pORI198 in transformed *L. lactis* cells was verified by PCR using primers M13F/M13R that could amplify the entire cloned region of the pSMA198 replication backbone. Untransformed *L. lactis* cells were used as a negative control. Stability of pORI198 in *L. lactis* (C). The stability of plasmid pORI198 was assessed as described in the manuscript. No statistically significant differences were observed between *L. lactis* cells grown in GM17 in the presence and the absence of erythromycin (p>0.05), indicating a 100% stability during a 100-generation time.

In order to experimentally assess the possibility of an ancestral genetic exchange event between *S. macedonicus* and *L. lactis* concerning pSMA198, we constructed the pORI198 *E. coli*—*S. macedonicus* shuttle vector based on the replication backbone of pSMA198 (Fig. 6A) and we tested the ability of this plasmid to replicate in *L. lactis* MG1363. Indeed, plasmid pORI198 was successfully cloned in the latter host (Fig. 6B). Interestingly, the plasmid was 100% stable in *L. lactis* for up to 100 generations, since no statistically significant difference was observed between cells that could grow in GM17 in the presence and absence of erythromycin (p>0.05, Fig. 6C). Furthermore, the PCN of pSMA198 in *S. macedonicus* and of pORI198 in *L. lactis* was low (2–3 copies per cell in both cases), suggesting that the pSMA198 backbone has equal replication efficiencies in the two hosts. The low copy number of the pSMA198 replicon is in agreement with the low copy number of other constructs based on the pCI305/pWV02 replicon [47]. The efficient replication of the pSMA198 replicon in *L. lactis* shows the full compatibility of this plasmid with this species, as well. This property coincides with the close phylogenetic relationship of the pSMA198 Rep protein to the respective proteins in dairy lactococcal pCI305/pWV02 plasmids mentioned above. Taken as a whole, our *in silico* analysis and our experimental findings provide strong evidence for the acquisition of pSMA198 by *S. macedonicus* from *L. lactis.* They also suggest that this acquisition took place in the dairy environment.

### The acquisition of pSMA198 by *S. macedonicus* seems not to be a recent event

As mentioned above, pSMA198 exhibits a high percentage of pseudogenes similarly to the chromosome of *S. macedonicus* ACA-DC 198 [16], suggesting that the two molecules must have co-evolved under the same gene decay processes.

**Figure 7:**
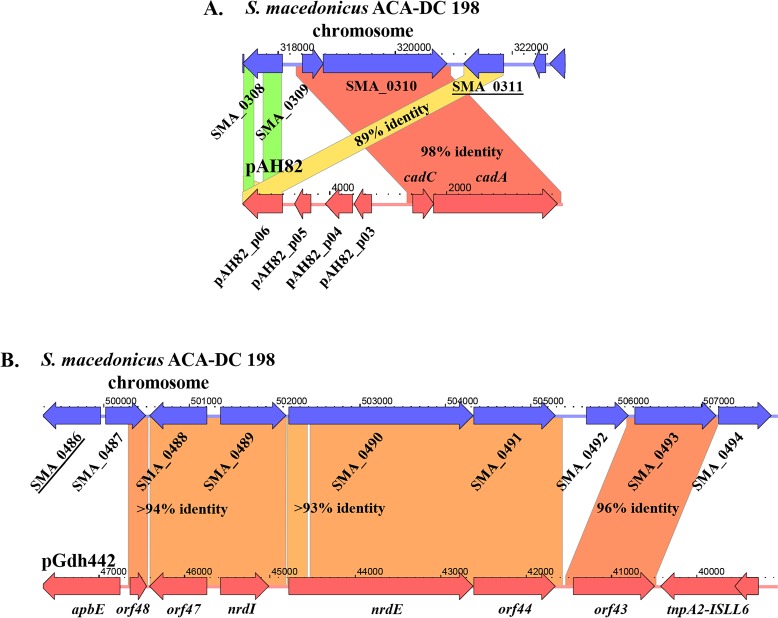
Sequence alignment of chromosomal regions of *S. macedonicus* ACA-DC 198 against pAH82 (A) and pGdh442 (B). The alignment was performed using Kodon software (Applied Maths, Belgium). In each case, the flanking transposase gene showing high sequence similarity to SMA_p0006 of pSMA198 plasmid is underlined. Colored areas between the sequences correspond to different levels of identity that are specified within the areas.

Given this inference on such a long co-existence, we examined possible genetic exchanges between pSMA198 and the chromosome of *S. macedonicus* ACA-DC 198. This hypothesis was also supported by the reduced size of pSMA198 as compared to some of its related plasmids, e.g. pCIS8 and pIL5 (12 kb vs. 80 kb and 23 kb, respectively) indicative of loss of genetic material, some of which could have moved to the chromosome. To identify such regions, we initially searched for the presence of chromosomal genes showing high identity to the genes found on pSMA198 or its related plasmids (Fig. 7). Using this strategy, a number of transposase-encoding genes were found on the chromosome that exhibited high sequence identity to the pSMA198 SMA_p0006 transposase pseudogene (e.g. SMA_0311 and SMA_0486, data not shown). Genes coding for similar transposases are also present in different lactococcal plasmids related to pSMA198 (e.g. pIL5, pCIS8, etc., data not shown). We then examined the genome of *S. macedonicus* for the existence of genes that could probably have derived from plasmids of the pCI305/pWV02 family in general. We found a 2.5 kb region within a large genomic island of *S. macedonicus* ACA-DC 198 containing genes SMA_0309 and SMA_0310 that showed ≥ 98% identity with *cadC* and *cadA* of pAH82. Plasmid pAH82 has been isolated from the dairy *L. lactis* subsp. *lactis* biovar diacetylactis DPC220 [48]. Noteworthy, the cassette of the cadmium resistance regulator (*cadC*) and the cadmium efflux ATPase (*cadA*) was previously suggested to have been also transferred horizontally from *L. lactis* to *S. thermophilus* [49]. An additional chromosomal region was found where five out of six genes (namely SMA_0488 to SMA_0493, involved in nucleotide biosynthesis and its regulation) showed ≥ 93% identity to the respective genes of plasmid pGdh442, which belongs to a plant isolate of *L. lactis* [50]. Strikingly, the two chromosomal regions (*cadC*-*cadA* and SMA_0488 to SMA_0493) are flanked at least on one side by one of the transposases showing high identity to pSMA198 SMA_p0006 mentioned above (i.e. SMA_0311 and SMA_0486). All these observations suggest a long co-existence with obvious genetic exchanges between the chromosome and the plasmid of *S. macedonicus*. It should be mentioned that a similar integration of plasmid sequences in the chromosome of *Streptococcus salivarius* has also been reported [51].

When looking at the distribution of pSMA198 in more than 10 randomly selected *S. macedonicus* strains of the ACA-DC collection, the plasmid was always present (data not shown). It should be emphasized that the strains belonged to different biotypes according to PFGE analysis and were all isolated from traditional Greek dairy products (our unpublished results). The widespread distribution of pSMA198 among *S. macedonicus* strains was also supported by its detection in strains from the Nestlé culture collection isolated in Switzerland (data not shown). Again, these findings suggest that the acquisition of pSMA198 from *S. macedonicus* is not a recent event. This seemingly universal presence of pSMA198 in strains of *S. macedonicus* and its potential acquisition in the milk environment from *L. lactis* led us to investigate if the plasmid plays a role in the growth of *S. macedonicus* in milk. Unfortunately, stress- or antibiotic-induced curing of the plasmid was not possible, presumably due to its high replication stability.

## Conclusion

Our findings demonstrate that pSMA198 is a novel member of the pCI305/pWV02 family of theta-replicating plasmids. The pCI305/pWV02 replicon has been shown to be of narrow host range, mainly replicating in *Lactococcus* species [37,46]. pSMA198 is the first streptococcal plasmid to be described within this family. The levels of sequence identity of the pSMA198 replication/mobilization backbone reflect an evolutionary history linked with different lactococcal plasmids supporting that *S. macedonicus* acquired pSMA198 from *L. lactis* and that this exchange took place most probably in milk or milk products. Indeed, the closest related plasmids to pSMA198 (i.e. pSK11b, pCIS8 and pIL5) have been isolated from *L. lactis* of dairy origin, while the pSMA198 Rep showed high phylogenetic relatedness to RepB proteins of *L. lactis* dairy isolates. The possibility that *L. lactis* may have acted as a donor of the pSMA198 plasmid is not surprising, since the species has been characterized as promiscuous to genetic exchange due to owning conjugative plasmids [44]. Finally, the acquisition of pSMA198 by *S. macedonicus* ACA-DC 198 seems not to be a recent event. This conclusion is based on: i) the high proportion of pseudogenes found in both pSMA198 and the chromosome of *S. macedonicus* ACA-DC 198, suggesting co-evolution of the two molecules, ii) the detection of different putative genetic exchange events between the two replicons and iii) the high prevalence of pSMA198 in divergent strains of *S. macedonicus.*

Our analyses thus point towards the long presence of *S. macedonicus* in the milk environment. This is in full agreement with the fact that most *S. macedonicus* strains (including *S. macedonicus* ACA-DC 198) have been isolated from dairy products that according to the current literature seem to be their primary ecological niche [6,16]. It should be noted that a plasmid related to lactoccocal plasmids has been also found in *S. infantarius* CJ18, the only other member of the SBSEC that was isolated from a dairy product [12]. Similar plasmids were absent from the *S. gallolyticus* and *S. pasteurianus* genomes sequenced to date that were all clinical isolates. Whether the presence of *S. macedonicus* ACA-DC 198 in the dairy environment has resulted in a diminished pathogenic potential compared to other streptococci, as suggested in some relevant studies [15,16,52–54], remains to be elucidated.

## Supporting Information

S1 Fig Maximum likelihood tree of the pSMA198 RepB generated using the Phylogeny.fr pipeline [27] and as described in the Materials and Methods section. The arrow indicates the position of the pSMA198 Rep and the bracket denotes the branch with its closest related proteins. Branch support values are presented in the tree, while branches showing <80% support were collapsed.
(PDF)
