# Supplementary material for: Acquisition through Horizontal Gene Transfer of Plasmid pSMA198 by Streptococcus macedonicus ACA-DC 198 Points towards the Dairy Origin of the Species
Source: PLoS One. 2015 Jan 13;10(1):e0116337. doi: 10.1371/journal.pone.0116337 (PMC4293149; doi:10.1371/journal.pone.0116337)
Supplement: S1 Fig — The arrow indicates the position of the pSMA198 Rep and the bracket denotes the branch with its closest related proteins. Branch support values are presented in the tree, while branches showing <80% support were collapsed. (PDF) [file pone.0116337.s001.pdf]

Figure S1

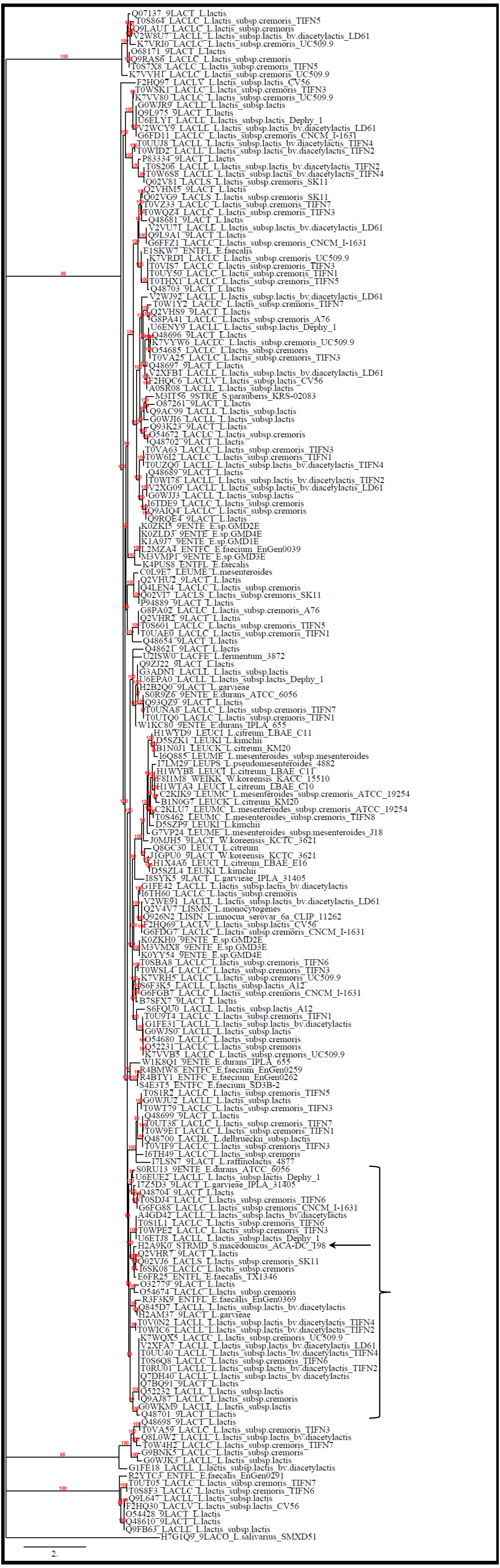

Maximum likelihood tree of the pSMA198 RepB generated using the Phylogeny.fr pipeline [27] and as described in the Materials and Methods section. The arrow indicates the position of the pSMA198 Rep and the bracket denotes the branch with its closest related proteins. Branch support values are presented in the tree, while branches showing <80% support were collapsed.
